# The *Non-Coding RNA* Journal Club: Highlights on Recent Papers—6

**DOI:** 10.3390/ncrna4030023

**Published:** 2018-09-18

**Authors:** Hua Xiao, Patrick K. T. Shiu, Jun Shu, Gaetano Santulli, Mohammad K. Gheybi, Simon J. Conn, Baptiste Bogard, Florent Hubé, Joseph H. Taube, Sendurai A. Mani, Luo Song, George A. Calin, Shuxing Zhang

**Affiliations:** 1 Division of Biological Sciences, University of Missouri, Columbia, MO 65211, USA; xiaohu@missouri.edu; 2 Department of Medicine, Einstein College of Medicine, Montefiore University Hospital, New York, NY 10461, USA; jun.shu@einstein.yu.edu; 3 Flinders Centre for Innovation in Cancer, Flinders University, Adelaide 5042, Australia; mohammadkazzem.gheybi@flinders.edu.au; 4 School of Pharmacy and Medical Sciences, University of South Australia, Adelaide 5000, Australia; 5 CNRS UMR7216, Epigenetics and Cell Fate, Université Paris Diderot, Sorbonne Paris Cité, 75013 Paris, France; baptiste.bogard@univ-paris-diderot.fr; 6 UMR7216 Epigénétique et Destin Cellulaire, Bâtiment Lamarck B, Case Courrier 7042, 35 rue Hélène Brion, 75013 Paris, France; 7 Department of Biology, Baylor University, Waco, TX 76706, USA; Joseph_Taube@baylor.edu; 8 Department of Translational Molecular Pathology, The University of Texas MD Anderson Cancer Center, Houston, TX 77030, USA; 9 Department of Experimental Therapeutics, The University of Texas MD Anderson Cancer Center, Houston, TX 77054, USA; luo.song@uth.tmc.edu (L.S.); gcalin@mdanderson.org (G.A.C.); 10 The University of Texas Graduate School of Biomedical Sciences, Houston, TX 77054, USA

## 1. Introduction

We are delighted to share with you our sixth Journal Club and highlight some of the most interesting papers published recently. We hope to keep you up-to-date with non-coding RNA research works that are outside your study area. The *Non-Coding RNA* Scientific Board wishes you an exciting and fruitful read.

## 2. Special Delivery from Plants to Pathogens

### Highlight by Hua Xiao and Patrick K. T. Shiu

Although it is known that hosts can send small RNAs (sRNAs), to pathogens, to inhibit their virulence, the mechanism of this transfer is unclear. In a recent issue of *Science*, Qiang Cai and coworkers showed that extracellular vesicles are involved in the process [1].

In this study, the authors purified fungal (*Botrytis cinerea*) cells from infected *Arabidopsis* tissues and identified 42 host-originated sRNAs (most of which could also be detected in extracellular vesicles isolated from apoplastic fluids). In animals, microRNAs are transferred via exosomes, which are derived from multivesicular bodies (MVBs). Tetraspanins (TET) are exosome markers in mammals, and *Arabidopsis* TET8 accumulates at the infection sites. At these sites, MVBs fuse with the plasma membrane to release vesicles (which can be readily uptaken by fungal cells), further supporting the notion that plants secrete exosomes to deliver sRNAs to their pathogens. Half of the transferred sRNAs have predicted targets, with a bias towards vesicle-trafficking pathways (which are important for fungal virulence). The target genes are down-regulated after an infection, unless the plant host is deficient in trans-acting small interfering RNA (tasiRNA) production. The cleavage of mRNAs could be detected in the fungus, suggesting that the transferred sRNAs silence the target genes through a transcript destruction.

This work showed that plant sRNAs are delivered to fungal cells via pathogen-induced exosomes. Future studies should shed light on how these defensive sRNAs are selectively packaged and transported and if this weapon delivery system is universal in cross-kingdom RNA interference (RNAi).

## 3. Connecting Myofibroblasts and Cardiomyocytes via Extracellular Vesicle-Associated microRNA: A Short-Distance Affair

### Highlight by Jun Shu and Gaetano Santulli

The expression of the small ubiquitin-like modifier 1 (SUMO1) has been reported to be significantly reduced in both human and murine heart failure (HF). However, the molecular mechanisms underlying SUMO1 upregulation had not been investigated, hitherto. Changwon Kho and colleagues identified a specific microRNA (miR) as a key factor in the pathophysiology of HF [2]. The authors demonstrated that miR-146a is a *SUMO1*-targeting miR, in failing human and mouse hearts. In a model of pressure overload, overexpression of miR-146a reduced SUMO1 expression, whereas miR-146a inhibition normalized SUMO1 expression and improved the cardiac function. Intriguingly, the authors demonstrated that miR-146a was not directly produced by cardiomyocytes, but it was first synthesized by activated fibroblasts (myofibroblasts), following an injury, and then transferred via extracellular vesicles to cardiomyocytes. This discovery has major implications in the clinical scenario, since targeting miR-146a might provide a novel therapeutic approach for the treatment of HF.

## 4. Size-Dependent Export of Circular RNAs from the Nucleus

### Highlight by Mohammad K. Gheybi and Simon J. Conn

While circular RNAs are co-transcriptionally synthesized in the nucleus, they are almost exclusively sequestered into the cytoplasm. This suggests circular RNAs (circRNAs), like other RNAs, are actively exported from the nucleus. In a recent issue of *Genes & Development*, Chuan Huang and co-workers illuminated a size-dependent mechanism of nuclear transport for circRNAs [3].

Using a targeted, small-interfering RNA (siRNA) screen against proteins involved in nuclear RNA export in *Drosophila*, the authors identified an RNA helicase—Hel25E—which caused nuclear retention of nascent circRNAs, longer than 800 nt. However, depletion of the principal mRNA nuclear export factor, the NXF1:NXT1 complex, had no quantifiable effect on circRNA transport. Extrapolating these results to human cells, targeting the two Hel25E homologs in HeLa cells, URH49 (DDX39A), and UAP56 (DDX39B), resulted in a nuclear retention of short (<356 nt) and long (>1298 nt) circRNAs, respectively.

This study identified the catalytic ATPase and helicase domains, of these human RNA helicases, as critical factors in defining circRNA export-size preference. Future studies could illuminate factors contributing to the nuclear export of intermediate-sized circRNAs and the possible involvement of distinct nucleoporins within the nuclear pore complex.

## 5. A New Bifunctional RNA at the Origin of Tumor Metastasis

### Highlight by Baptiste Bogard and Florent Hubé

PNUTS (Phosphatase 1 Nuclear Targeting Subunit) was originally identified as a modulator of the PP1 phosphatase on Retinoblastoma protein (Rb). PNUTS is up-regulated in cancer cells where it acts as a potential oncogene through sequestration of the tumor-suppressor PTEN in an inactive state, in the nucleus.

Grelet and colleagues identified an alternatively spliced isoform of PNUTS mRNA generated through an alternative acceptor splice site, in exon 12. The resulting frame-shift disrupted the translation capacity of the transcript, hence producing a long non-coding RNA (lncRNA-PNUTS) [4]. The authors identified heterogeneous nuclear ribonucleoprotein (hnRNP) E1 as being required to prevent alternative splicing of PNUTS precursor RNA and favor the formation of the PNUTS mRNA. They showed that the lncRNA-PNUTS acts as a sponge for miR-205, a negative regulator of genes involved in epithelial-to-mesenchymal transition (EMT), limiting its bioavailability. They tested the consequences on tumor progression and showed a temporary increase in the expression of the ZEB proteins, a family of transcription factors that regulate EMT, which in turn trigger downstream EMT events.

This study reported an example of a mammalian lncRNA—produced by an originally protein-coding gene—through alternative splicing, which is involved in the early stages of EMT and possibly cancer progression.

## 6. Screening for Long Non-Coding RNA Mediators of Chemotherapy Resistance

### Highlight by Joseph H. Taube and Sendurai A. Mani

Forward genetic screens have yielded fantastic insights into gene-phenotype relationships, for decades. Bester and colleagues have adopted this strategy to identify long non-coding RNAs (lncRNAs), as well as protein-coding genes, which, when over-expressed, confer altered sensitivity to Ara-C, a deoxycytidine analog used to treat acute myeloid leukemia [5]. As lncRNAs exert their function through both trans and cis mechanisms, endogenous loci, rather than transgenes, were activated using a CRISPRa-SAM [6]. This was achieved by linking a deactivated Cas9 to the VP64 co-activation domain, expressing a synthetic co-activator, and then applying a library of aptamer-tagged single guide RNA (sgRNA) specific to the known lncRNA and coding gene promoters. 

The authors identified hundreds of candidate lncRNAs, either associated with Ara-C sensitization, or resistance. They further confirmed the role of several lncRNA, including the GAS6 antisense transcript (GAS6-AS2). GAS6-AS2 was further shown to act in cis mechanisms to upregulate the expression of its cognate protein-coding gene (GAS6), and in trans mechanisms to alter the DNA methylation at *AXL*. Validating this strategy, a higher expression of GAS6-AS2, as well as AC008073.2, were associated with poor prognosis and decreased disease-free survival, in acute myeloid leukemia (AML) patients treated with Ara-C. This approach showed the power of forward genetic approaches to capture key regulators among lncRNA-encoding genes.

## 7. Long Non-Coding RNA “Entrap” Tumor Suppressor microRNAs to Promote Cancer Growth and Migration

### Highlight by Luo Song, George A. Calin and Shuxing Zhang

We have a deep interest in modeling RNA–protein [7], RNA–RNA [8], and RNA–small molecule interactions [9]. Such profundity will significantly help us elucidate novel molecular mechanisms of tumorigenesis and provide insights for therapeutics discovery and development. Herein, we are delighted to share one of the interesting papers we published recently on ncRNA–ncRNA interactions [8].

The transcribed ultraconserved regions (T-UCRs) had been proven to play an important role in human carcinogenesis, but there is still a large blank in the mechanisms and the consequences of their expression dysregulation in cancers. Recently, our colleagues revealed a new pathway of the transcribed ultraconserved region 339 (*uc.339*) to explain how *uc.339* promotes carcinogenesis [8].

In this article, the authors assessed the related data from The Cancer Genome Atlas (TCGA) database and found that, in 210 non-small cell lung cancer (NSCLC) patients, the high expression of *uc.339* was positively correlated with the low survival. Furthermore, the authors showed that with upregulation of the transcribed *uc.339* in archival NSCLC samples, tumor suppressor microRNAs, such as miR-339-3p, -663b-3p, and -95-5p, were suppressed, which led to an increased level of a cell-cycle regulation protein, cyclin E2. As a result, cancer growth and migration was promoted. Additionally, the result suggested that either overexpression or suppression of these microRNAs had limited effects on the *uc.339* level. Based on their modeling studies, the authors named this type of interaction as “entrapping”. This study further revealed structural details of how lncRNAs play roles in human carcinogenesis, and also provided a novel concept for cancer therapy and drug discovery.
